# From belief to identity: teacher resilience as a mediating mechanism between growth mindset and professional identity among EFL student teachers

**DOI:** 10.3389/fpsyg.2026.1830864

**Published:** 2026-05-29

**Authors:** Amal Mubarak Alsuwaylim, Mohammed A. Alshehri, Ali Lamouchi, Mohamed Sayed Abdellatif, Ashraf Ragab Ibrahim, Mohamed Ali Nemt-allah

**Affiliations:** 1 Department of Psychology, College of Education, Prince Sattam bin Abdulaziz University, Al-Kharj, Saudi Arabia; 2 Department of Curriculum and Instruction, College of Education, King Saud University, Riyadh, Saudi Arabia; 3 Department of English Language and Literature, College of Science and Humanities, Prince Sattam bin Abdulaziz University, Al-Kharj, Saudi Arabia; 4 Educational Psychology and Statistics Department, Faculty of Education, Al-Azhar University, Dakahlia, Egypt

**Keywords:** EFL student teachers, growth mindset, mediation analysis, professional identity, teacher education, teacher resilience

## Abstract

Professional identity development among EFL student teachers represents a critical yet psychologically demanding process, shaped by competing pressures including language anxiety, practicum challenges, and emotional labor. Despite growing recognition of growth mindset as a pivotal psychological resource in teacher education, the mechanisms through which it influences professional identity remain insufficiently understood. This study examines the mediating role of teacher resilience in the relationship between growth mindset and professional identity among EFL student teachers. A quantitative cross-sectional survey design is employed with a main sample of 706 EFL student teachers. Data are collected using three validated instruments: the Growth Mindset Scale, the Vietnam Teachers' Resilience Scale, and the Teacher Professional Identity Scale. Mediation analysis is conducted using Hayes' PROCESS macro with 5,000 bootstrap samples and 95% bias-corrected confidence intervals. Results reveal that growth mindset is significantly associated with both teacher resilience and professional identity. Teacher resilience was statistically consistent with a partial indirect role, accounting for 28.55% of the total association. These findings, drawn from a convenience sample of Egyptian EFL student teachers and therefore limited in generalizability, highlight the complementary roles of growth mindset and resilience in shaping professional identity, within similar Arabic-speaking institutional contexts.

## 1 Introduction

Professional identity, defined as the evolving sense of “who I am as a teacher,” constitutes the foundational core of a teacher's career development and serves as a central organizing framework for instructional decision-making, role interpretation, and classroom behavior (Alam et al., 2024; Cheng, 2021; Li, 2023; Ma, 2022). For EFL student teachers, this self-concept is dynamically constructed through accumulated experiences, beliefs, values, motivations, and pedagogical competencies, continuously negotiated through interactions with students, colleagues, and institutional contexts (Le et al., 2023; Ostad et al., 2019; Wu, 2022). A coherent and well-developed professional identity is strongly associated with heightened commitment, sustained work engagement, reduced burnout, and greater instructional effectiveness in the language classroom (Ma, 2022; Ostad et al., 2019; Xing, 2022; Zhu et al., 2025).

Despite its centrality, the professional identity of EFL student teachers remains particularly vulnerable to a constellation of interconnected pressures. Language anxiety and the inherent tension of occupying dual roles as both learner and teacher simultaneously generate persistent feelings of inadequacy and professional insecurity (Ardi et al., 2023; Kudaibergenov and Lee, 2020; Orlova and Kamenická, 2024). Practicum experiences further expose student teachers to the stark gap between idealized teaching and classroom realities, where managing diverse proficiency levels, student misbehavior, and institutional demands frequently triggers identity crises and self-doubt (Karimi et al., 2021; Wu et al., 2025). Compounding these challenges, the intensive emotional labor inherent in language teaching, including the sustained suppression of negative emotions to support anxious learners, significantly heightens burnout risk and destabilizes emerging professional identities (Gkonou and Miller, 2017, 2020; Li and Liu, 2021; Shen, 2022; Yetkin and Alagözlü, 2025).

Amid these pressures, growth mindset has emerged as a pivotal psychological resource for EFL student teachers' professional development. Defined as the belief that teaching abilities, including lesson design, classroom management, and language instruction, are malleable and improvable through sustained effort, effective strategies, and receptiveness to feedback (Lin et al., 2022; Liu et al., 2023; Yeager et al., 2021), a growth mindset fundamentally reframes professional challenges as developmental opportunities rather than evidence of fixed inadequacy (Bardach et al., 2024; Laine and Tirri, 2023; Ronkainen et al., 2019). EFL student teachers who embrace this belief demonstrate greater work engagement, perseverance, and occupational wellbeing during demanding practicum experiences (He et al., 2023; Liu et al., 2023; Meierdirk and Fleischer, 2022), while also exhibiting more constructive responses to supervisory feedback, instructional setbacks, and classroom failures (Lou and Noels, 2020; Yeager et al., 2021).

Empirical and theoretical scholarship has increasingly established a meaningful relationship between growth mindset and professional identity development among student teachers. Because professional identity is itself understood as inherently dynamic, continuously negotiated, and shaped through experience and reflection rather than as a fixed trait (Flores, 2020; Keiler, 2018; Rushton et al., 2023), it aligns conceptually with the growth mindset conviction that professional competencies remain perpetually improvable (Lin et al., 2022; Luo et al., 2025; Zhu et al., 2025). Student teachers who internalize this belief interpret practicum difficulties as integral to becoming a teacher rather than as evidence of permanent professional inadequacy (Flores, 2020; Zungu and Santos, 2025), while simultaneously sustaining the self-efficacy, motivation, and psychological need satisfaction that consolidate a coherent, positive professional identity (Cheng, 2021; Lin et al., 2022; Luo et al., 2025; Sun et al., 2025).

Within this demanding landscape, teacher resilience emerges as an indispensable psychological capacity for navigating the inherent stressors of EFL practicum experiences. Defined as the ability to bounce back from setbacks, recover emotional strength, and adapt positively when confronting adversity and professional challenges (Chu and Liu, 2022; Gu et al., 2024; Zhang, 2021), resilience is linked to student teachers' maintain psychological wellbeing, sustain commitment, and avoid burnout despite recurring difficulties in lesson delivery, classroom management, and institutional expectations (Duan et al., 2023; Han, 2022; Li, 2023; MacIntyre et al., 2020; Zaimoglu and Dagtaş, 2025). Critically, resilience functions as a dynamic, learnable quality rather than a fixed trait, shaped through reflective practice, institutional support, and contextual interactions (Beltman and Poulton, 2019; Pozo-Rico et al., 2023; Sairattanain, 2025; Xue, 2021; Zhang and Luo, 2023).

Theoretical and empirical evidence suggests that growth mindset is theoretically and empirically associated with teacher resilience by fundamentally reframing how student teachers interpret and respond to classroom failures. When student teachers believe their teaching abilities are malleable rather than fixed, setbacks are cognitively reappraised as valuable learning opportunities signaling “not yet mastered” rather than as definitive proof of professional inadequacy (Anderson et al., 2021; Bardach et al., 2024; Dweck and Yeager, 2019; Janapati and Vijayalakshmi, 2024). This cognitive reframing promotes adaptive attributions to controllable factors such as effort and strategy selection, sustains task-specific self-efficacy amid adversity, and encourages flexible problem-solving approaches that collectively provide the mental stamina and persistence essential for bouncing back from inevitable practicum difficulties (Lee et al., 2023; Lin et al., 2022; Lou and Noels, 2020; Meierdirk and Fleischer, 2022). Resilience is prioritized as the focal mediator because, unlike self-efficacy (task-specific confidence), grit (dispositional persistence), or emotion regulation (discrete affective management), it uniquely captures adaptive recovery across repeated adversarial episodes, positioning it as the most proximal bridge between growth mindset beliefs and professional identity consolidation.

The hypothesized mediating role of teacher resilience between growth mindset and professional identity finds theoretical grounding in an integrated framework combining Dweck's Growth Mindset Theory, Bandura's Social Cognitive Theory, Fredrickson's Broaden-and-Build Theory, and Identity Process Theory (Bardach et al., 2024; Dweck and Yeager, 2019; Lin et al., 2022; Tang et al., 2025; Yeager and Dweck, 2020). These perspectives are integrated sequentially rather than additively: growth mindset supplies the belief-level input, social cognitive theory explains resilience-building through self-efficacy, broaden-and-build theory accounts for resource expansion, and identity process theory specifies identity consolidation. Partial mediation is expected because growth mindset directly implicates identity malleability independently of resilience (Calo et al., 2024; Liu, 2025; Siu et al., 2025; Tang et al., 2025). This accumulated resilience, in turn, enables student teachers to integrate challenging practicum experiences into a coherent, positive professional self-concept, ultimately strengthening their professional identity (Luo et al., 2025; Samsudin et al., 2025; Yang et al., 2021; Zhang et al., 2024). Despite this growing scholarship, no prior study has empirically examined teacher resilience as the specific psychological mechanism linking growth mindset to professional identity among Arabic-speaking EFL student teachers, leaving this indirect pathway untested. The present study addresses this gap directly.

This study aims to examine the relationship between growth mindset and professional identity among EFL student teachers, with particular attention to the potential indirect role of teacher resilience in this relationship. Specifically, the study seeks to investigate whether growth mindset was significantly associated with teacher resilience and professional identity, whether teacher resilience was significantly associated with professional identity, and whether teacher resilience is consistent with a significant indirect variable in the relationship between growth mindset and professional identity. Based on the theoretical framework and empirical evidence reviewed, it is hypothesized that growth mindset was associated with both a direct positive relationship on professional identity and an indirect association operating through teacher resilience, such that higher growth mindset levels were linked to greater resilience, which in turn was associated with stronger professional identity among EFL student teachers.

## 2 Method

### 2.1 Study design

This study employed a quantitative cross-sectional survey design to examine the mediating role of teacher resilience in the relationship between growth mindset and professional identity among EFL student teachers. The cross-sectional approach was deemed appropriate given that the study aimed to capture participants' perceptions and self-reported experiences at a single point in time rather than tracking changes longitudinally. This design is consistent with prior research investigating psychological constructs and their interrelationships among student teachers in educational contexts.

### 2.2 Participants

Participants were recruited from the Faculties of Education and Humanities at Al-Azhar University and Kafr El-Sheikh University, Egypt. All participants were Arabic-speaking EFL student teachers enrolled in undergraduate English language teacher preparation programs. Both samples were drawn from the same two institutions and the same target population of undergraduate EFL student teachers during the same data collection period. Independence between samples was ensured by assigning participants to either the psychometric or the main sample prior to data collection, using a non-overlapping allocation procedure, so that no participant appeared in both samples.

The study involved two independent samples drawn using convenience sampling. A psychometric sample (*N* = 586) was used to evaluate the factorial validity and reliability of the adapted instruments, while a main sample (*N* = 706) was used to test the hypothesized structural model. Participants in the psychometric sample were EFL student teachers whose ages ranged from 19 to 22 years (*M* = 20.53, SD = 1.08), enrolled across four undergraduate grade levels. In the main sample, participants' ages similarly ranged from 19 to 22 years (*M* = 20.47, SD = 1.14). Further demographic details for both samples are presented in Table 1.

**Table 1:** Demographic characteristics of study participants.

Variable	Category	Psychometric sample (***N*** = 586)		Main sample (***N*** = 706)	
		*N*	%	*N*	%
Gender	Male	187	31.9	226	32.0
	Female	399	68.1	480	68.0
Age	19	130	22.2	191	27.1
	20	154	26.3	168	23.8
	21	165	28.2	169	23.9
	22	137	23.4	178	25.2
Grade level	First	147	25.1	181	25.6
	Second	160	27.3	150	21.2
	Third	140	23.9	173	24.5
	Fourth	139	23.7	202	28.6

*N*, frequency; %, valid percentage of total sample.

### 2.3 Measures

The Growth Mindset Scale (GMS; Sigmundsson and Haga, 2024) is an 8-item unidimensional instrument measuring the overall growth mindset construct, with items rated on a 5-point Likert scale ranging from 1 (not like me at all) to 5 (very much like me). Confirmatory factor analysis conducted on the psychometric sample yielded excellent model fit (CMIN/DF = 0.594, GFI = 0.995, CFI = 0.998, RMSEA = 0.020). Reliability estimates were equally strong, with McDonald's omega and Cronbach's alpha both exceeding 0.92 (ω = 0.924, α = 0.929). The CMIN/DF value of 0.594 reflects a well-fitting, parsimonious model and, while below 1.0, is mathematically permissible and interpretable as indicating that the model does not overfit the data; this value has been verified against the original AMOS output.

The Vietnam Teachers' Resilience Scale (VITRS; Trang and Thang, 2023) is a 20-item instrument comprising four dimensions: social resilience, professional resilience, emotional resilience, and motivational resilience, with 5 items per dimension rated on a 5-point Likert scale ranging from 1 (strongly disagree) to 5 (strongly agree). CFA results supported good model fit (CMIN/DF = 1.013, GFI = 0.973, CFI = 0.966, RMSEA = 0.035). Reliability estimates were satisfactory at both the subscale level (ω ranging from 0.882 to 0.895) and for the total scale (ω = 0.931, α = 0.930). For the VITRS, both the four-factor first-order model and a higher-order model with a single resilience factor were tested and compared. The higher-order model demonstrated acceptable fit (CMIN/DF = 1.089, GFI = 0.971, CFI = 0.963, RMSEA = 0.037) and was retained, justifying the use of a composite total score in subsequent analyses.

The Teacher Professional Identity Scale (TPIS; Wong and Liu, 2022) is an 18-item instrument organized across three dimensions: teacher self-efficacy (9 items), commitment to teaching (4 items), and professional orientation (5 items), with items rated on a 5-point Likert scale; teacher self-efficacy items are rated from 1 (not well at all) to 5 (very well), while commitment to teaching and professional orientation items are rated from 1 (strongly disagree) to 5 (strongly agree). CFA results demonstrated excellent model fit (CMIN/DF = 1.185, GFI = 0.971, CFI = 0.997, RMSEA = 0.018). Reliability was strong at both the subscale level (ω ranging from 0.854 to 0.928) and for the total scale (ω = 0.963, α = 0.963). Similarly, for the TPIS, the three-factor first-order structure and a higher-order model were both evaluated. The higher-order solution yielded excellent fit (CMIN/DF = 1.201, GFI = 0.969, CFI = 0.995, RMSEA = 0.019), supporting aggregation of subscales into a total professional identity score.

### 2.4 Procedures

Data were collected using self-report questionnaires administered via Google Forms over a 2-week period from October 15 to 29, 2025, during the first semester of the 2025/2026 academic year. All instruments were originally developed in English and were translated into Arabic, the participants' native language, followed by back-translation into English to ensure linguistic equivalence and conceptual accuracy. Participation was voluntary, and all participants were assured of the confidentiality of their responses prior to completing the survey.

Translation was performed by two bilingual academics with doctoral-level expertise in educational psychology and English linguistics. Back-translation was independently conducted by a third bilingual scholar with no prior exposure to the original instruments. Following back-translation, a panel of five subject-matter experts reviewed all items for semantic equivalence, cultural appropriateness, and conceptual clarity. Minor wording adjustments were made to 3 items in the VITRS and 2 items in the TPIS to better reflect the Arabic-speaking EFL student teacher context; no items were removed. A pilot study involving 30 EFL student teachers (not included in either the psychometric or main samples) was conducted prior to full data collection. Participants were asked to flag any items perceived as ambiguous or culturally unfamiliar, and cognitive interviewing confirmed adequate item comprehension across all three instruments.

### 2.5 Data analysis

Descriptive statistics and bivariate correlations were computed using SPSS version 27, while confirmatory factor analysis was performed using AMOS version 26 to evaluate the psychometric properties of all three instruments with the psychometric sample. The hypothesized mediation model was subsequently tested using Hayes' PROCESS macro (Model 4) with 5,000 bootstrap samples and 95% bias-corrected confidence intervals to estimate direct, indirect, and total effects. Mediation was inferred when the bootstrap confidence interval for the indirect effect excluded zero, indicating a statistically significant indirect pathway from growth mindset to professional identity through teacher resilience. Although CFA was employed to validate instrument structure, mediation analysis utilized composite scores within Hayes' PROCESS macro rather than latent variable modeling. This approach was retained for three reasons: (1) higher-order CFA confirmed strong unidimensional representation of each construct, supporting composite aggregation; (2) all instruments demonstrated excellent reliability (ω > 0.92), minimizing measurement error concerns; and (3) PROCESS macro remains widely adopted in teacher education mediation research for its interpretive accessibility.

Although both the VITRS and TPIS are multidimensional instruments, total composite scores were used in the mediation model for three reasons: (1) higher-order CFA confirmed that a single overarching factor adequately represented the variance in each instrument's subscales; (2) the theoretical constructs of resilience and professional identity are treated as holistic psychological resources in the guiding frameworks; and (3) the primary research question concerns the overall indirect association between growth mindset and professional identity rather than dimension-specific pathways. Harman's single-factor test revealed that the first unrotated factor accounted for 31.4% of total variance, below the 50% threshold, suggesting common method variance did not critically distort the findings, though this test remains a conservative diagnostic rather than a definitive remedy.

## 3 Results

Prior to testing the hypothesized mediation model, descriptive statistics and normality indicators were examined for all study variables. As presented in Table 2, the means for Growth Mindset, Teacher Resilience, and Professional Identity suggest that participants reported moderately high levels across all three constructs. Skewness and kurtosis values for all variables fell within the acceptable range of ±2, indicating that the data approximated a normal distribution and were suitable for parametric analyses.

**Table 2:** Descriptive statistics for study variables.

Variable	Min	Max	M	SD	Skewness	Kurtosis
Growth mindset	8.00	40.00	31.49	5.30	−0.37	−0.16
Teacher resilience	31.00	100.00	76.47	12.70	−0.22	−0.25
Social resilience	10.00	25.00	19.12	3.26	−0.22	−0.34
Professional resilience	8.00	25.00	19.14	3.30	−0.23	−0.28
Emotional resilience	7.00	25.00	19.10	3.35	−0.19	−0.30
Motivational resilience	6.00	25.00	19.11	3.32	−0.22	−0.12
Professional identity	29.00	90.00	71.50	11.02	−0.45	−0.05
Teacher Self-efficacy	16.00	45.00	35.74	5.56	−0.41	−0.16
Commitment to teaching	6.00	20.00	15.88	2.61	−0.38	−0.24
Professional orientation	7.00	25.00	19.89	3.21	−0.48	0.12

*M*, mean; SD, standard deviation.

Preliminary independent-samples *t*-tests and one-way ANOVAs revealed no statistically significant differences in growth mindset, teacher resilience, or professional identity scores across gender or age groups. A marginally significant difference was observed across grade levels for professional identity (*F* = 2.81, *p* = 0.039), with fourth-year students scoring slightly higher, consistent with accumulated practicum experience.

To examine the bivariate relationships among the study variables, Pearson correlation coefficients were computed. As presented in Table 3, Growth Mindset demonstrated a significant positive correlation with Teacher Resilience (*r* = 0.652, *p* < 0.01) and Professional Identity (*r* = 0.764, *p* < 0.01), while Teacher Resilience was significantly and positively correlated with Professional Identity (*r* = 0.673, *p* < 0.01), supporting the theoretical rationale for the proposed mediation model.

**Table 3:** Pearson correlation matrix for study variables.

Variable	1	2	3	4	5	6	7	8	9	10
1. Growth mindset	1									
2. Teacher resilience	0.652^**^	1								
3. Social resilience	0.669^**^	0.896^**^	1							
4. Professional resilience	0.659^**^	0.901^**^	0.892^**^	1						
5. Emotional resilience	0.646^**^	0.895^**^	0.900^**^	0.889^**^	1					
6. Motivational resilience	0.684^**^	0.961^**^	0.960^**^	0.959^**^	0.960^**^	1				
7. Professional identity	0.764^**^	0.673^**^	0.672^**^	0.670^**^	0.670^**^	0.699^**^	1			
8. Teacher Self-efficacy	0.740^**^	0.636^**^	0.635^**^	0.635^**^	0.628^**^	0.660^**^	0.898^**^	1		
9. Commitment to teaching	0.743^**^	0.651^**^	0.644^**^	0.643^**^	0.640^**^	0.671^**^	0.921^**^	0.875^**^	1	
10. Professional orientation	0.777^**^	0.679^**^	0.677^**^	0.675^**^	0.673^**^	0.704^**^	0.985^**^	0.944^**^	0.962^**^	1

^**^p < 0.01 (two-tailed).

Inter-subscale correlations among TPIS dimensions ranged from *r* = 0.875 to *r* = 0.985, suggesting limited discriminant validity. Similarly, Motivational Resilience showed exceptionally high correlations with other resilience dimensions (*r* = 0.959–0.961). Accordingly, only total composite scores are interpreted in subsequent analyses.

**Figure 1:**
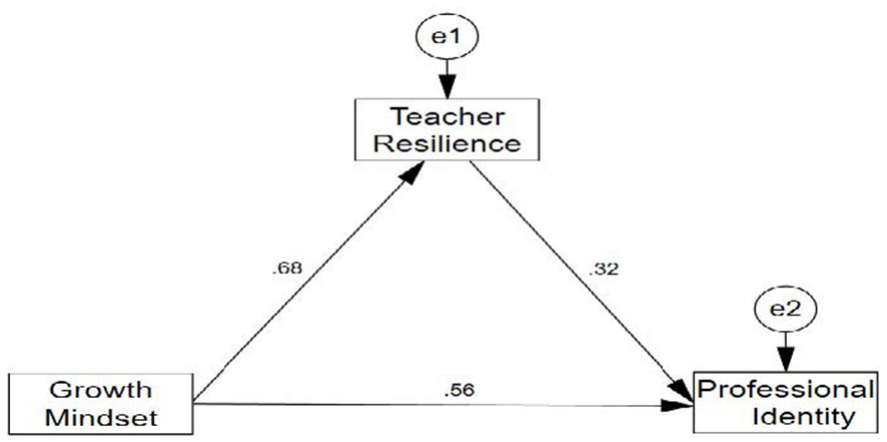
Mediation model illustrating the role of teacher resilience as a mediator in the relationship between growth mindset and professional identity.

The hypothesized mediation model was tested using Hayes' PROCESS macro (Model 4) with 5,000 bootstrap samples and 95% bias-corrected confidence intervals. The model examined the role of Teacher Resilience as a mediator in the relationship between Growth Mindset (GMS) and Professional Identity. The overall model accounted for a substantial proportion of variance in both Professional Identity (*R*^2^ = 0.660) and Teacher Resilience (*R*^2^ = 0.468), both statistically significant. A visual representation of the model with all standardized path coefficients is presented in Figure 1, while detailed path statistics are reported in Table 3.

As presented in Table 4, Growth Mindset was significantly and positively associated with Teacher Resilience (β^*^ = 0.684, *p* < 0.001), and Teacher Resilience, in turn, was significantly associated with Professional Identity (β^*^ = 0.324, *p* < 0.001). Growth Mindset also maintained a significant direct association with Professional Identity after controlling for Teacher Resilience (β^*^ = 0.556, *p* < 0.001), suggesting that the indirect effect is partial rather than complete.

**Table 4:** Coefficients for the proposed Indirect-effects model.

Path	β^*^	β	SE	*T*	*p*	95% bootstrap CI
GMS → VITRS	0.684^**^	1.637	0.066	24.87	< 0.001	[1.508, 1.767]
GMS → TPIS	0.556^**^	1.154	0.063	18.41	< 0.001	[1.031, 1.277]
VITRS → TPIS	0.324^**^	0.282	0.026	10.75	< 0.001	[0.230, 0.333]

GMS, Growth Mindset Scale; VITRS, Vietnam Teachers' Resilience Scale; TPIS, Teacher Professional Identity Scale. β^*^, standardized coefficient; β, unstandardized coefficient; SE, standard error; CI, confidence interval. ^**^p < 0.001.

The decomposition of total, direct, and indirect effects is summarized in Table 5. The indirect effect of Growth Mindset on Professional Identity through Teacher Resilience was statistically significant (β^*^ = 0.222, β = 0.461, 95% Boot CI [0.368, 0.562]), accounting for 28.55% of the total effect. Since both the direct effect (71.45% of total) and the indirect effect were significant and their bootstrap confidence intervals excluded zero, Teacher Resilience is consistent with a partial mediator pattern in the association between Growth Mindset and Professional Identity among EFL student teachers.

**Table 5:** Total, direct, and indirect effects of growth mindset on professional identity.

Effect	β^*^	β	SE	*T*	*P*	95% bootstrap CI	% of total
Total effect (GMS → TPIS)	0.777	1.615	0.049	32.74	< 0.001	[1.518, 1.711]	100%
Direct effect (GMS → TPIS)	0.556	1.154	0.063	18.41	< 0.001	[1.031, 1.277]	71.45%
Indirect effect (GMS → VITRS → TPIS)	0.222	0.461	0.049	—	—	[0.368, 0.562]	28.55%

β^*^, standardized coefficient.

## 4 Discussion

The findings of this study reveal that growth mindset was significantly associated with both teacher resilience and professional identity among EFL student teachers, with teacher resilience consistent with a partial indirect role in this relationship. The overall model explains 66.0% of the variance in professional identity. Whilst substantial, this figure should be interpreted cautiously given potential conceptual overlap between professional identity's self-efficacy subscale and the predictor constructs, which may partially inflate explained variance estimates. These findings should be interpreted within the specific institutional and cultural context of Egyptian EFL teacher education programs, and caution is warranted when extrapolating to broader EFL populations.

The indirect association pathway through teacher resilience represented 28.55% of the total association, while the direct effect remained substantial at 71.45%. These results are consistent with the hypothesized indirect-effects model and suggest that although growth mindset was directly associated with how student teachers perceive and construct their professional selves, a meaningful portion of this association was attributable to the psychological mechanism of resilience, which was in turn linked to more adaptive responses to the inherent stressors of EFL practicum contexts. These findings advance prior literature by identifying teacher resilience as a specific, quantifiable psychological mechanism linking growth mindset to professional identity, a pathway previously theorized but not empirically tested within Arabic-speaking EFL student teacher contexts.

These findings align with and extend a growing body of empirical and theoretical scholarship. The significant relationship between growth mindset and professional identity corroborates prior work emphasizing that professional identity is a dynamic, negotiated construct inherently compatible with beliefs about malleability and improvement (Flores, 2020; Lin et al., 2022; Luo et al., 2025; Zhu et al., 2025). The positive association between growth mindset and resilience is consistent with evidence that reframing setbacks as learning opportunities sustains self-efficacy and adaptive coping amid adversity (Bardach et al., 2024; Dweck and Yeager, 2019; Lou and Noels, 2020; Meierdirk and Fleischer, 2022). Furthermore, the mediating role of resilience in connecting inner psychological resources to identity outcomes resonates with frameworks proposed by Chu and Liu (2022), Li (2023), and Yang et al. (2021), reinforcing resilience as a critical bridge between cognitive dispositions and professional self-concept among student teachers.

This interpretation is further supported by Daniilidou et al. (2025), whose cluster analysis identified distinct high- and lower-resilience teacher profiles associated with self-efficacy, emotional intelligence, and burnout, reinforcing the conceptualization of resilience as a dynamic, multidimensional construct shaped by psychological resources rather than fixed traits. Additionally, Boczkowska et al. (2024) demonstrated that resilience protective factors vary across demographic and institutional contexts, highlighting the importance of social and personal competencies in sustaining teacher resilience, a finding consistent with the partial mediation pattern observed in the present study.

While causal conclusions cannot be drawn from the present cross-sectional data, the observed associations tentatively suggest several directions for EFL teacher preparation that warrant experimental verification. Concretely, programs could incorporate structured reflection activities where student teachers reframe instructional setbacks as developmental milestones, with mindset shifts tracked through pre-post assessments. Resilience-building components, including peer mentoring sessions and emotional regulation workshops, could be evaluated using validated resilience scales across practicum stages. Supervisory feedback protocols could be redesigned to emphasize effort-based attributions, with outcomes measured through professional identity scores at successive practicum points. Given that resilience partially mediates the growth mindset-professional identity relationship, addressing both constructs concurrently is likely to yield stronger professional identity outcomes than targeting either in isolation. These recommendations are provisional; their effectiveness as program or policy interventions requires confirmation through longitudinal or experimental designs before implementation can be confidently advocated.

Several limitations warrant careful consideration when interpreting the present findings. First, the cross-sectional design precludes causal inferences, as directionality among growth mindset, resilience, and professional identity cannot be established from a single measurement point. Second, common-method bias remains a concern despite Harman's single-factor test and supplementary marker variable analysis; notably, the more rigorous common latent factor procedure could not be implemented because Hayes' PROCESS macro operates on composite scores rather than item-level indicators, structurally precluding CLF estimation. Third, findings apply primarily to Arabic-speaking EFL student teachers in Egyptian institutional contexts, limiting broader generalizability. Fourth, although instruments underwent rigorous translation and back-translation, full cultural adaptation cannot be assumed, and measurement invariance across Arabic-speaking contexts requires future validation. Fifth, demographic variables were examined descriptively but not incorporated as covariates, potentially introducing uncontrolled confounding. Finally, treating resilience as a unitary mediator may obscure differential effects across its four constituent dimensions.

Future research should prioritize longitudinal designs tracking developmental trajectories of growth mindset, resilience, and professional identity across successive practicum experiences, thereby enabling stronger causal conclusions. Experimental and quasi-experimental studies evaluating mindset intervention programs would provide more rigorous evidence for the pathways identified here. Critically, future studies should employ full latent-variable structural equation modeling with item-level or parcel-level indicators, which would permit implementation of the common latent factor procedure for more definitive common method bias assessment—a diagnostic structurally precluded in the present composite-score framework. Researchers should additionally explore whether the four resilience dimensions function as distinct mediators with differential strengths, and whether demographic variables such as gender or grade level moderate the hypothesized chain. Cross-cultural comparative studies and mixed-methods approaches incorporating qualitative narratives would further clarify boundary conditions and enrich understanding of how student teachers psychologically navigate these processes.

## 5 Conclusion

This study offers preliminary correlational evidence consistent with teacher resilience functioning as a meaningful partial indirect variable in the theoretically informed association between growth mindset and professional identity development among EFL student teachers. By suggesting that beliefs about the malleability of teaching abilities may be associated with higher psychological resilience, this research underscores the importance of integrating both constructs within EFL teacher preparation. The substantial direct effect further suggests that growth mindset independently associates with professional identity beyond its resilience-building function. These associations, observed within a specific Egyptian EFL context, tentatively suggest that growth mindset and teacher resilience may serve as complementary psychological resources among comparable student teacher populations, supporting coherent professional identities needed for long-term effectiveness. Causal conclusions, however, cannot be drawn from the present cross-sectional data, and experimental or longitudinal designs are needed before these findings can confidently inform program or policy decisions.

## Data Availability

The raw data supporting the conclusions of this article will be made available by the authors, without undue reservation.
